# Bispecific antibodies as powerful immunotherapeutic agents for urological cancers: Recent innovations based on preclinical and clinical evidence

**DOI:** 10.7150/ijbs.96155

**Published:** 2025-01-27

**Authors:** Kiavash Hushmandi, Behzad Einollahi, E Hui Clarissa Lee, Reo Sakaizawa, Antonino Glaviano, Russel J. Reiter, Seyed Hassan Saadat, Marzieh Ramezani Farani, Yun Suk Huh, Amir Reza Aref, Shokooh Salimimoghadam, Alan Prem Kumar

**Affiliations:** 1 Nephrology and Urology Research Center, Clinical Sciences Institute, Baqiyatallah University of Medical Sciences, Tehran, Iran.; 2 Department of Pharmacology, Yong Loo Lin School of Medicine, National University of Singapore, Singapore, Singapore.; 3 NUS Center for Cancer Research (N2CR), Yong Loo Lin School of Medicine, National University of Singapore, Singapore, Singapore.; 4 Department of Biological, Chemical and Pharmaceutical Sciences and Technologies, University of Palermo, 90123 Palermo, Italy.; 5 Department of Cell Systems and Anatomy, UT Health San Antonio, Long School of Medicine, San Antonio, Texas USA.; 6 NanoBio High-Tech Materials Research Center, Department of Biological Sciences and Bioengineering, Inha University, 100 Inha-ro, Incheon 22212, Republic of Korea.; 7 Department of Vitro Vision, DeepkinetiX Inc., Boston, MA, USA.; 8 Department of Biochemistry and Molecular Biology, Faculty of Veterinary Medicine, Shahid Chamran University of Ahvaz, Ahvaz, Iran.

**Keywords:** Urologic neoplasms, Bispecific antibodies, Immunotherapy, Drug delivery

## Abstract

Conventional immunotherapy has emerged as a key option for cancer treatment. However, its efficacy has been limited in urological cancers, especially prostate cancer, because of the immunosuppressive tumor microenvironment (TME), difficulty in drug delivery, aberrant immune response, and damage to normal cells. Bispecific antibodies (BsAbs) are engineered proteins with two different antigen-binding domains, designed using different technologies and in various formats. BsAb-based tumor immunotherapy has yielded optimistic results in preclinical and clinical investigations of many tumor types, including urological cancers. However, a series of challenges, including tumor heterogeneity, TME, Ab immunogenicity, adverse effects, serum half-life, low response rates, and drug resistance, hamper the application of BsAbs. In this review, we provide insights into the most common BsAb platforms with different mechanisms of action, which are under preclinical and clinical research, along with ways to overcome the challenges in BsAb administration for treating urological cancer.

## 1. Background

Common urological cancers include prostate, kidney, bladder, testis, and penile cancers. While prostate, testicular, and penile cancers occur only in men, kidney and bladder cancers can affect both men and women, with men being more susceptible. Many novel therapeutic strategies have been introduced in recent years to manage cancer and increase the survival of patients; however, treating these cancer types, especially when they acquire a metastatic state, remains challenging, particularly in prostate carcinoma 1-3.

In recent decades, therapeutic antibodies (Abs) have been developed as important components of cancer treatment because of their specificity and sensitivity 4. Approved in 1986 by the Food and Drug Administration (FDA), muromonab is considered the pioneer of monoclonal Ab (mAb)-based therapeutic strategies 5. Despite the success of mAbs in cancer therapy, various factors and pathways are involved in cancer pathophysiology. Thus, achieving the desired goal is difficult because of drug resistance and toxicity 6-8. The introduction of bispecific antibodies (BsAbs) has addressed the problem of drug resistance and improved the efficacy of cancer therapy 9-11. BsAbs recognize two distinct antigens or antigenic epitopes and target specific points; they also redirect effector T cells and other immune cells to the tumor site 12. While most BsAbs under development are used to treat cancers, others are focused on chronic inflammatory conditions, hematological disorders, and infectious diseases 13.

The concept of BsAbs was initially proposed by Nisonoff *et al.* in 1961 and was based on coupling the Fab fragments of two different rabbit antibodies using reoxidation 14. In 1975, Milstein and Kohler introduced hybridoma technology by fusing splenic B lymphocytes and myeloma cells of immunized mice to solve the problem of producing pure mAbs 15. In 1983, Milstein *et al.* succeeded in producing the first BsAb with an IgG structure by fusing two different hybridoma cells, known as hybrid-hybridoma (quadroma) technology 16. In 1985, Perez *et al.* identified the anti-tumor function of BsAbs, which could bind to T cells and tumor-associated antigens (TAA), resulting in T cell recruitment to the malignant site and tumor killing 17. In 1988, Huston *et al.* developed a single-chain variable fragment (scFv), with fewer refolding problems 18. Emergence of the knobs-into-holes technology in 1996 significantly advanced BsAb production 19. With improvements in biotechnology and antibody engineering, various platforms were developed and BsAbs gradually entered marketing. In 2009, the European Medicines Agency (EMA) approved catumaxomab as the first therapeutic BsAb in Europe. Catumaxomab targets CD3 and the epithelial cell adhesion molecule (EpCAM), and is used to treat patients with malignant ascites; however, it was withdrawn from the market in 2017 owing to commercial reasons (July 10, 2017; EMA (428877/2017). However, it is still under investigation. Since 2014, the FDA has approved nine BsAbs for treating cancer and hematologic and ocular diseases.

In 2014, the FDA approved blinatumomab, an antibody targeting CD3 and CD19, which was developed for treating acute lymphoblastic leukemia. Emicizumab, amivantamab, tebentafusp, faricimab, teclistamab, mosunetuzumab, epcoritamab, and glofitamab are other BsAbs, approved by the FDA since 2021, mostly for treating hematologic cancers and disorders 20.

Considering the promising and effective results from the clinical application of BsAbs, they have become a top issue in Ab drug research. Recently, more than 200 clinical and preclinical studies have been reported in the field of BsAbs.

Therapeutic options available for urological cancer treatment show limited efficacy, and the development of novel and more specific therapies is a medical necessity. Several preclinical and clinical studies have evaluated the anti-tumor effects of BsAbs against urological cancers. Most of these studies provide valuable data and warrant further investigation in larger cohorts. This review describes the different types and mechanisms of action of BsAbs, advances in BsAb design, and the preclinical and clinical trial developments of BsAbs in urological cancers. In addition, it discusses the challenges and potential solutions for BsAb-based therapy, and future perspectives.

## 2. The landscape of bispecific antibodies

A conventional antibody (Ab) is a dual-valent but monospecific molecule comprising two antigen-binding (Fab) segments and one crystallizable fragment (Fc), which together form the characteristic Y configuration. Bispecific antibodies (BsAbs) that target two separate antigens can overcome the constraints of monospecific Abs, leading to reduced adverse effects and lower frequency of administration 21,22. However, the greater variety and complexity of BsAb structures compared with those of mAbs necessitate more sophisticated techniques for their synthesis and refinement 23,24. The BsAb sector is advancing rapidly and numerous structural variations are being explored. Initially, BsAbs were synthesized using quadroma technology; however, with the advent of genetic engineering, recombinant DNA methodologies have become paramount for crafting BsAbs with diverse attributes such as size, penetrability, serum longevity, and valency 25,26.

Based on the presence of the Fc region, BsAbs are typically classified into two groups: IgG-like (IgG-based) and non-IgG-like (fragment-based) (**Table 1**).

### 2.1. IgG-like BsAbs

IgG-like BsAbs resemble standard antibodies as they possess an Fc region and are composed of complete IgG molecules. The presence of an Fc region offers numerous benefits, including enhanced solubility and stability owing to improved purification, extended serum half-life, and involvement in immune effector functions, such as antibody-dependent cell-mediated cytotoxicity, antibody-dependent cellular phagocytosis, and complement-dependent cytotoxicity 40,41. However, these BsAb variants have drawbacks such as off-target binding of the active Fc region to Fc receptors (FcRs), which can lead to adverse effects and issues related to chain association 42. Coexpression of two distinct heavy (H) and light (L) chains poses initial challenges in BsAb development, reducing the likelihood of producing a functional BsAb from an array of potential combinations 43. Strategies have been devised recently to address these challenges. The main IgG-like BsAb formats include Triomab quadroma, Knobs-into-holes, CrossMab, Orthogonal, Duobody, XmAb, DVD-Ig, and FIT-Ig.

The Triomab quadroma approach relies on the somatic fusion of two disparate hybridomas, yielding a structure similar to that of classical Abs with two unique antigen-binding sites. However, this method is prone to mispairing the H and L chains. Creation of a chimeric rat/mouse quadroma mitigates this issue 27,42. For example, catumaxomab is a BsAb comprising murine IgG2a anti-CD3 and rat IgG2b anti-EpCAM 44.

Despite modifications to the quadroma platform, heavy-chain mispairing challenges have persisted. The knobs-into-holes technique, which involves engineering the CH3 domain of each H chain, was developed to minimize mismatches and enhance Fc heterodimerization 45. This strategy, which entails replacing a smaller amino acid with a larger one to create a 'knob' and vice versa to form a 'hole,' has resolved the issue of H chain mismatches; however, the L chain problem remains 29. Solutions such as the use of a common L chain, additional mutations in the VH-VL and CH-CL interfaces, and CrossMab formatting have been proposed to address this issue 30,46.

The CrossMab format, derived from the knobs-into-holes concept, involves domain swapping within one Fab of an Ab (CrossMabVH-VL) or exchanging the CL domain of the L chain with the CH1 domain of the H chain (CrossMabCH1-CL), thereby ensuring proper chain pairing 47,48. Faricimab, a CrossMab-based BsAb that targets ang-2 and VEGF, is currently FDA-approved for treating diabetic macular edema and nAMD 42.

The Orthogonal Fab interface, which introduces mutations into the variable region of Fab, allows the production of BsAbs in mammalian cells 31,49. LY3164530, which targets epidermal growth factor receptor (EGFR) and c-MET, is an example of this format 50.

The Duobody platform, achieved through controlled Fab arm exchange (cFAE), consistently produces the desired BsAb with high efficiency 42,51,52. Amivantamab, which targets MET and EGFR, is designed for the treatment of advanced non-small cell lung cancer and is based on this format 53,54.

The XmAb platform, developed by Moore *et al.*, incorporates four mutations at the CH3-CH3 interface to enhance heavy chain heterodimerization, thus increasing FcR affinity and the potential for immune cell activation, as well as improving serum half-life through FcRn binding 33,55. XmAb20717, which targets PD-1 and CTLA-4, is currently in phase 2 clinical trials for advanced prostate cancer (NCT05032040).

Tetravalent BsAbs such as DVD-Ig and FIT-Ig, with four antigen-binding sites for two different targets, have also been developed. DVD-Ig features two variable domains per Fab arm, linked by short, flexible connectors, ensuring proper H and L chain pairing 34,56,57. Lutikizumab, a DVD-Ig-based BsAb, targeting IL-1α and IL-1β, is in phase 2 clinical trials for treating knee osteoarthritis synovitis 58.

FIT-Ig, comprising two sets of Fab domains, was introduced in 2017 36. EpimAb, an FIT-Ig BsAb, targets EGFR and c-MET for treating advanced/metastatic solid tumors 59.

### 2.2. Non-IgG-like BsAbs

This category of BsAbs lacks the Fc segment and comprises variable light and heavy domains from two antibodies, or Fab units, linked with connectors such as disulfide or non-covalent bonds 60,61. Non-IgG-like BsAbs are characterized by their straightforward design and benefits including small size, reduced immunogenicity, cost-effectiveness, high production yield, enhanced tumor infiltration, and resolution of chain-related complications 26,46. However, they exhibit a brief serum lifespan, necessitating frequent dosing. Further, they are prone to reduced structural integrity and a heightened risk of aggregation, which are notable drawbacks.

Among these, the bispecific T cell engager (BiTE), also referred to as tandem scFv, is a BsAb with two scFv regions from two monoclonal antibodies connected by a supply peptide linker. One scFv in BiTE is invariably directed against CD3, whereas the other targets a tumor-associated antigen (TAA), facilitating the linkage of CD3^+^ T cells with specific tumor cells without the need for costimulation signals 62,63.

Blinatumomab is the inaugural FDA-sanctioned BiTE molecule 62,63. Additionally, a CD3 × prostate-specific membrane antigen (PSMA), BiTE BsAb was developed to treat PSMA-expressing tumors 64. Owing to the ephemeral half-life of BiTE BsAbs (approximately 2-4 hours), an advanced BiTE variant was conceived by combining BiTE with the Fc region, thereby prolonging its serum half-life to seven days. CD3 × CD19 half-life extended BiTE (HLE-BiTE) (AMG562) has demonstrated efficacy in treating CD19^+^ malignancies 65,66.

Dual-affinity retargeting molecules (DARTs) are synthesized by linking the VL and VH (scFv) domains of one antibody with those of another to form heterodimeric polypeptide chains 38. DARTs surpass BiTEs in terms of stability and efficacy in triggering T-cell responses against cancer cells. For instance, flotetuzumab, a DART targeting CD3ϵ and CD123, is accessible in Japan and Europe for treating refractory acute myeloid leukemia 67. Integration of the Fc fragment into DART, termed DART-Fc, enables FcRn-mediated recycling of the molecule 68.

Tandem diabodies (TandAbs) are quadrivalent entities comprising two peptide chains arranged inversely, each bearing two antigen-binding sites for distinct antigens (in the VL-1, VH-2, VL-2, and VH-1 configurations). TandAbs offer greater stability than that of their predecessors and can redirect T and NK cells to tumors 26,69. AFM11, which targets CD3 and CD19, has shown promising results in constraining Raji tumors *in vivo* 70. Furthermore, AFM13, another TandAb that binds to CD30 and CD16A on NK cells, has been utilized for the treatment of Hodgkin's lymphoma 71.

The bi-nanobody architecture consists solely of a heavy chain devoid of light chains. By connecting the VH domains of two or more antibodies, a bi- or multi-specific antibody is formed 39. This framework is notable for its simplicity, robustness, and superior tissue penetration; however, it suffers from a short serum half-life, which can be mitigated by conjugation with serum albumin 26,72. Ozoralizumab, a bi-nanobody-based BsAb featuring two anti-TNFα nanobodies and one anti-HAS nanobody, is currently undergoing phase 2 clinical trials for treating rheumatoid arthritis 72.

## 3. Mechanisms of action and design principles of bispecific antibodies

### 3.1. Mechanisms of action of BsAbs

**Figure 1:**
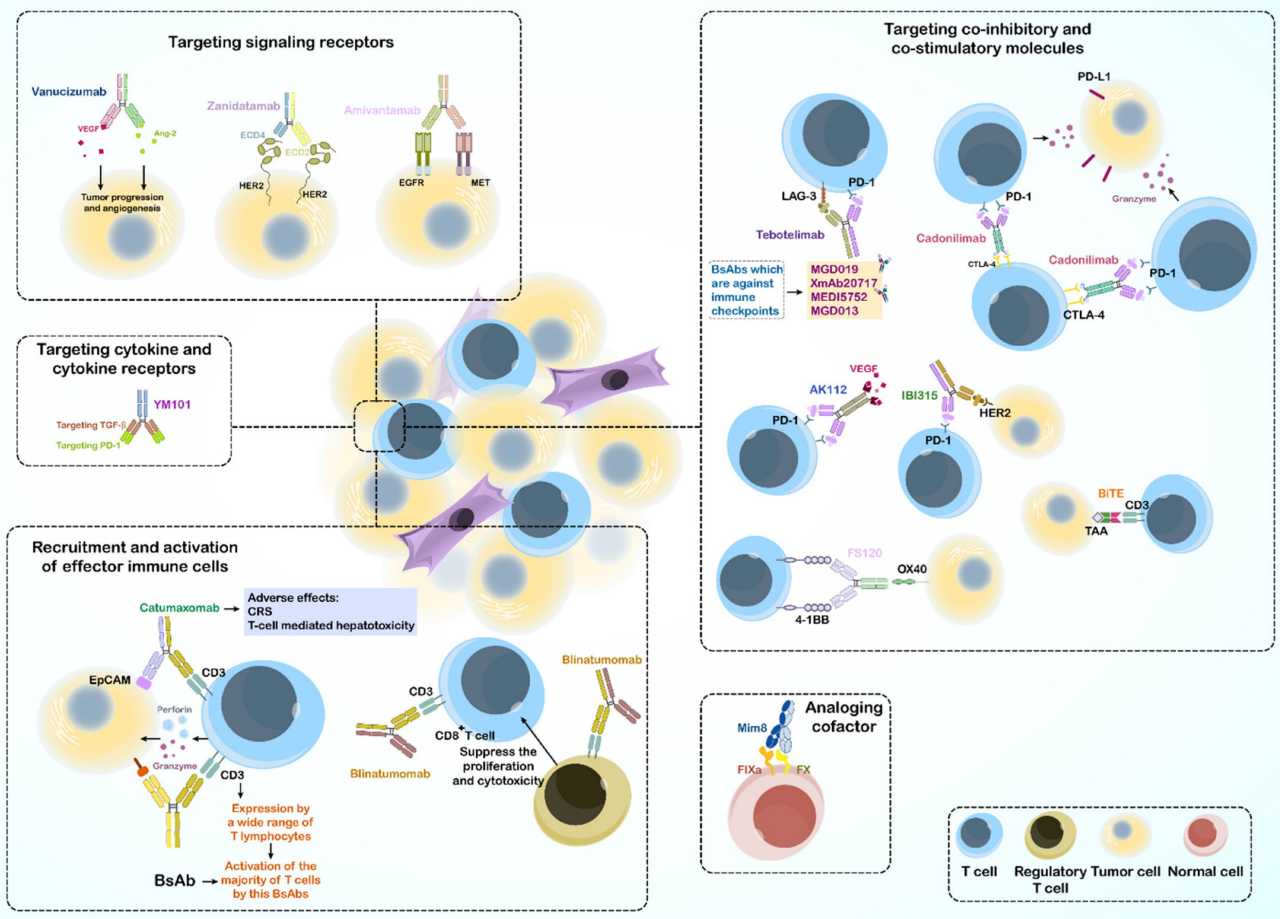
Bispecific antibodies: Mechanisms of action*.*

Targeting of two different antigens by BsAbs provides a variety of functional pathways; thus, BsAbs are used to treat diseases through multiple mechanisms. Some BsAbs play a role in recruitment and activation of innate and adaptive immune cells. Accordingly, they can connect immune cells with tumor cells to kill the tumor. Some BsAbs suppress co-inhibitory receptors, whereas others activate costimulatory molecules to activate immune cells. Other mechanisms include activation or inhibition of signaling receptors, targeting cytokines and their receptors, analogous cofactors, and using a target to transport them (**Figure 1**).

#### 3.1.1. Recruitment and activation of effector immune cells

The initial application of BsAbs in cancer treatment focused on T cells recruitment to the site of tumor T cell-engaging BsAbs (T-BsAbs), which can connect T cells to specific cancer cells and ultimately improve tumor killing 73,74.

Owing to the fact that BsAbs mostly activate T cells through binding of the ε subunit of CD3 in the TCR complex, they bypass the major histocompatibility complex (MHC) restriction and induce activation without antigen presentation 75. This characteristic also circumvents tumor evasion strategies such as loss of MHC expression or defects in antigen presentation 76.

Further, activated T cells can induce tumor cell necrosis or apoptosis by producing perforin and granzyme or stimulating death ligands, such as the Fas-FasL pathway 75. Blinatumomab induces cancerous cell removal in Philadelphia chromosome-negative B cell ALL by redirecting human T cells 77.

However, using BsAbs in cancer immunotherapy may also cause severe adverse effects like the cytokine release syndrome and cell T cell mediated cell cytotoxicity related to off-target binding of the active Fc domain to the FcγR of immune cells 78-81.

As CD3 is expressed by a wide range of T lymphocytes, CD3 binding can activate the majority of the T cell population, including regulatory T cells 82,83. An *in vitro* study reported that human regulatory T cells activated by blinatumomab suppressed the proliferation and cytotoxic effects of CD8^+^ cytotoxic T lymphocytes (CTLs) 84. Targeting specific T-cell subsets circumvents the activation of immunosuppressive regulatory T cells and decreases the risk of side effects.

Activation of cytotoxic γδ T cells was successful as BsAb was used to target the γδTCR and a human ovarian cancer antigen *in vitro* 85. 7D12-5 GS-6H4 is a novel BsAb targeting Vγ9 chain of the Vγ9Vδ2 TCR and of epidermal growth factor receptor (EGFR). 7D12-5 GS-6H4 induces activation of Vγ9Vδ2 T cells, a small subpopulation of γδ T cells, and increases apoptosis of colorectal cancer cells in a mouse xenograft model 86.

In addition to T cells, BsAbs can target specific antigens on the surface of NK cells [called Bispecific killer cell engager (BiKE)] and macrophages. For instance, AFM13, a TandAb that recognizes CD16A on NK cells and CD30 on cancerous lymphocytes, is used for treating Hodgkin's lymphoma, and is in phase I clinical trials 71. Overall, AFM13 can selectively activate NK cells and provide anti-tumor immunity 87.

#### 3.1.2. Targeting co-inhibitory and co-stimulatory molecules

Immune cells express multiple regulatory molecules on their surface, including a group of immune checkpoint proteins that regulate cell activation, proliferation, and anti-tumor activity 88. Stronger anti-tumor immunity is achieved by inhibiting or stimulating the relevant pathways 89,90.

In cancer immunotherapy using immune checkpoint inhibitors (ICIs), immune cell activation in the tumor microenvironment (TME) is enhanced, resulting in the apoptosis of tumor cells, making ICIs an important treatment option for cancer 91,92. The therapeutic idea of blocking two inhibitory immune checkpoints came from the promising results of combinational therapy. For example, a clinical trial demonstrated the 5-year outcomes of nivolumab (anti-PD-1) and ipilimumab (anti-CTLA-4) combination therapy in advanced melanoma, which resulted in long-term progression-free and overall survival compared to those with monotherapy using each of the Abs 93.

Tebotelimab (MGD013) is a tetravalent DART BsAb, targeting PD-1 and LAG-3 and is used to control solid tumors and hematologic malignancy in phase 1 clinical trials 94. Recent studies have shown that tebotelimab is more effective than PD-1 antibody monotherapy 95.

Cadonilimab is a tetrabody BsAb that targets both PD-1 and CTLA-4. While infiltrated lymphocytes in the TME showed increased co-expression of CTLA-4 and PD-1 compared with that in peripheral T cells, cadonilimab enrichment was enhanced in the TME to activate exhausted T cells. This BsAb has been used in China since 2022 for treating recurrent or metastatic cervical cancer (R/M CC) 96.

Other BsAbs targeting dual immune checkpoints include MGD019, XmAb20717, MEDI5752, and MGD013, which are dual-target 6.

Further, BsAbs can target immune checkpoints and TAA simultaneously, such as AK112 (PD1 ˟ VEGF) and IBI315 (PD1 ˟ HER2). The mechanism of action in some BsAbs is based on the combination of T-cell redirection and immune checkpoint blockade, such as an anti-CD33 ˟ CD3 BiTE, which links to the extracellular domain of PD-1, known as checkpoint inhibitory T-cell engager (CiTE). The CiTE Ab leads to enhanced T cell cytotoxic effects toward CD33+ PD-L1+ cancer cells in preclinical models of acute myeloid leukemia 6,97.

Co-stimulatory molecules expressed by T cells through the TCR, are critical for T cell activation, survival, proliferation, cytokine production, and release 98.

FS120 is a BsAb on a tetravalent platform that targets 4-1BB and OX40. FS120 activates the 4-1BB pathway and subsequently induces T-cell activation and proliferation. To increase specificity and reduce the side effects of FS120, the binding arm targeting 4-1BB is activated only after Ab binding to OX40 99,100. Further, many TAA-targeted 4-1BB-agonistic BsAbs, which are Fc-free or Fc-silenced, show acceptable anti-tumor activity without toxicity in mouse and primate models, and some of these are currently in clinical development 101-103.

#### 3.1.3. Targeting signaling receptors

Targeting two tumor receptors using BsAbs ensures specific binding of the Ab to tumor cells, even if one of the desired TAAs is also expressed by normal cells, and low toxicity is observed. Many studies have reported dysregulation of multiple signaling pathways and proteins in tumor cells. Thus, targeting them provides a better approach for treating and overcoming drug resistance.

##### 3.1.3.1. inhibition of signaling receptors

The inhibition of signaling pathways can suppress tumor development and angiogenesis in the TME. The HER family and VEGFR are two well-known receptor tyrosine kinases (RTKs) that play key roles in cell proliferation and the execution of cell programs 104. Mutations in RTKs and abnormal activation of their signaling in cells are associated with cancer development 105. Single targeting of RTKs in cancer seems to be effective in treatment; however, other RTKs in tumor cells can escape inhibition, resulting in drug resistance. Overall, BsAbs target multiple RTKs or their ligands and block two or more signaling pathways to reduce tumor cell escape.

Amivantamab, approved by the FDA on May 21, 2021, targets EGFR and MET simultaneously and circumvents drug resistance in non-small cell lung cancer 106-109.

Another example is a BsAb that recognizes two epitopes on the same antigen. Zanidatamab (ZW25) is a BsAb against two non-overlapping domains of HER2 antigen (ECD4 and ECD2) and has yielded promising results in treating HER2-positive breast cancer and gastroesophageal adenocarcinoma (GEA) in a phase 1 clinical trial 110,111. By targeting angiogenesis, vanucizumab provides a potential therapy for advanced solid tumors, as it targets VEGF and Ang-2, both of which are involved in tumor growth and angiogenesis 112.

##### 3.1.3.2. Activation of signaling receptors

In contrast to inhibition of the desired signaling by inhibitory BsAbs, some therapeutic processes require activation of receptor signaling using agonistic antibodies. This mechanism of action of BsAbs is useful for treating diseases other than cancer. BFKB8488A, a bispecific antibody against fibroblast growth factor receptor 1 (FGFR1) and KLB has been used to treat obesity-related metabolic defects by activating FGFR1/KLB 113,114.

#### 3.1.4. Targeting cytokine and cytokine receptors

Abnormal cytokine regulation is associated with tumor progression, and targeting these cytokines improves treatment efficacy. Although the high immunogenicity and short half-life of cytokine antagonists limit their use, combination therapies may overcome this limitation, which sparked the idea of BsAb designing 115,116.

A combination using TGF-β inhibitors with ICIs increases anti-tumor activity 117. For example, YM101, a BsAb targeting TGF-β and PD-1 based on the Checkbody platform, promoted the formation of hot tumor by increasing the attracting lymphocytes and dendritic cells to the tumor site, elevating the ratio of M1/M2, increasing cytokine production in T cells, and finally destroying the tumor 118.

In addition to cancer immunotherapy, BsAbs can also be used to treat inflammatory diseases such as rheumatoid arthritis. ABT122, designed in the DVD-Ig format, targets TNF-α and IL-17 to treat rheumatoid arthritis 119.

#### 3.1.5. Acting as analogous cofactors

BsAbs can also act as enzymes or as a cofactor for an enzyme. The Mim8 BsAb targets activating factor IX (FIXa) and factor X (FX), as an activated factor VIII (FVIII) mimetic. which is important for achieving hemostasis. This BsAb can be used to treat hemophilia A in patients with congenital factor VIII deficiency 120.

#### 3.1.6. Using target to transport

BsAbs can use the specificity of their first target to transport their second specific target, also known as hijacking. For example, in neurological diseases, BsAbs transport the transferrin receptor from their first antigen-binding domain across the blood-brain barrier (BBB) in immune-privileged brain regions and target pathogenic mediators with their second antigen-binding site 121,122. This process is also useful in treating viral and bacterial infection 123,124. Finally, targeting CD63 (also known as LAMP3), which is involved in lysosomal trafficking, and HER2, a TAA, using a BsAb can improves the intracellular delivery of an antibody-drug conjugate to the lysosome 125.

### 3.2. The design principle of BsAbs

**Figure 2:**
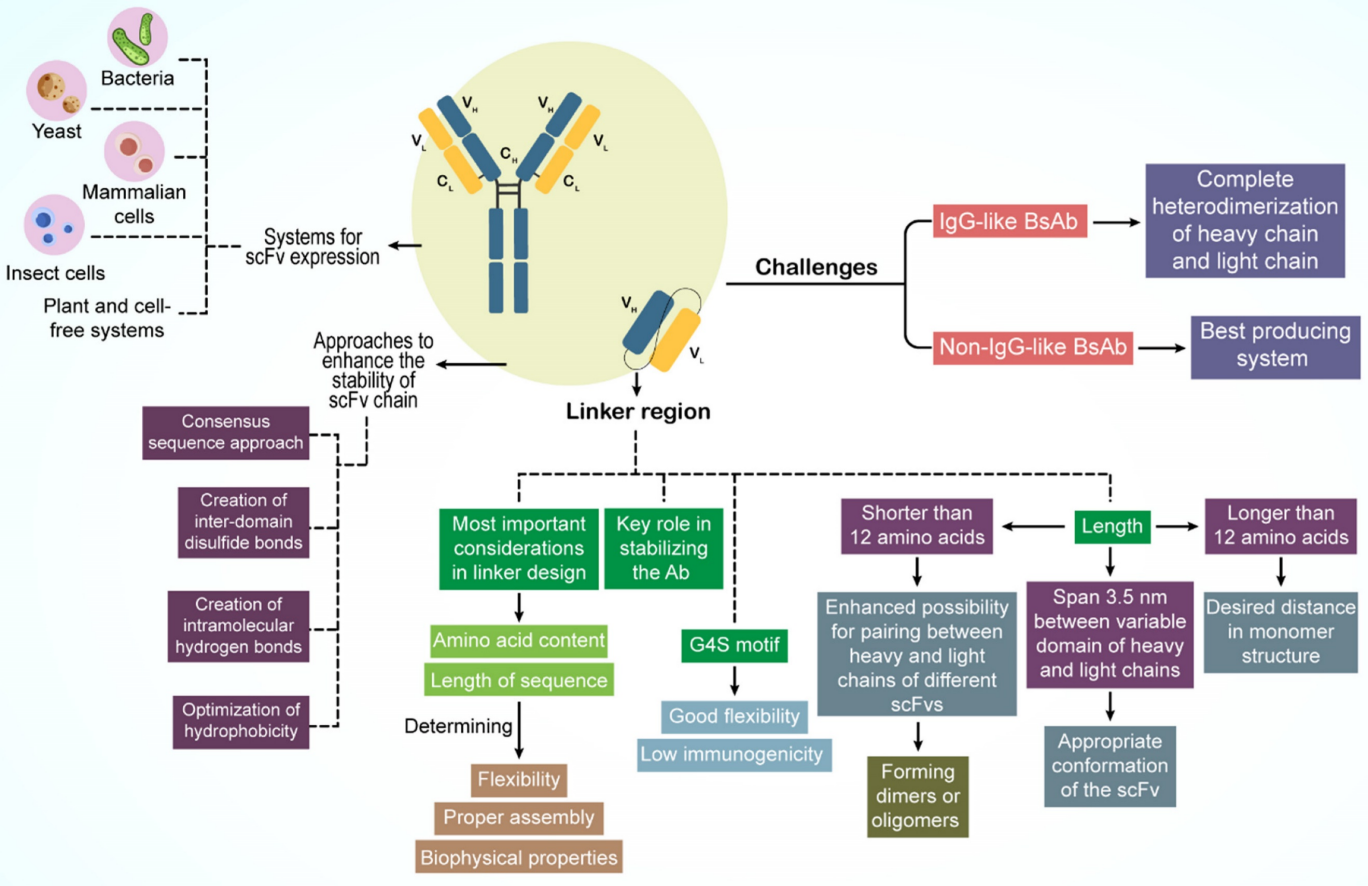
Design principles of BsAbs.

Creating a high-quality BsAb with minimal contamination is a significant hurdle. For non-IgG BsAbs, the optimal production system depends on factors such as BsAb size, amino acid sequence, protein configuration, solubility, stability, and purification processes. Conversely, for IgG-like BsAbs, complete heterodimerization of the heavy and light chains is crucial for the production of pure antibodies (**Figure 2**).

For non-IgG-like BsAbs, the linker region connecting the light and heavy chain domains is vital for antibody stabilization and scFv optimization 126. The composition and sequence length of the amino acids in the linker are pivotal; they govern the flexibility, assembly accuracy, and biophysical characteristics of BsAbs 26. The G4S motif (G: glycine; S: serine), a commonly used linker, demonstrates notable flexibility and minimal immunogenicity 60.

The linker length is critical, ideally extending 3.5 nm between the variable domains of the heavy and light chains to ensure that scFv adopts the correct structure 26. Moreover, scFv stability is essential for BsAb design, and directly influences the biological function of the antibody. Stable scFv regions also serve as foundational elements for BsAbs 127. Various methods exist to bolster scFv chain stability, such as the consensus sequence approach, in which a mutation is introduced into the most prevalent amino acid in the homologous Fv domain to enhance stability. Other techniques include the formation of interdomain disulfide bonds, intramolecular hydrogen bonds, and refinement of hydrophobic interactions 26.

Diverse platforms are available for the expression of non-IgG-like BsAbs, including bacterial, yeast, mammalian, insect, plant, and cell-free systems, each of which is suitable for different BsAbs 128,129. *Escherichia coli* is a favored host for scFv expression owing to its rapid growth and ease of genetic manipulation despite its lack of chaperones and post-translational modification processes, which necessitate further scFv modifications. In contrast, mammalian cells offer a promising alternative, providing protein folding pathways and post-translational modifications to prevent misfolding 129-131.

Addressing chain-associated issues is a primary concern for IgG-like BsAbs 23. Several strategies have been devised to modify the CH3 domain for proper heavy chain assembly, as detailed in Section 2.1. Advanced methods for enhancing heavy- and light-chain interactions are discussed in the same section.

An innovative solution to light-chain mispairing involves the production of BsAbs through the combination of half-antibodies from two distinct cell lines, a co-culture approach that reduces contamination risks and costs 132. This method involves inoculating two separate *E. coli* strains, each harboring a plasmid for one half antibody, into a single vessel, facilitating the production of a broader range of stable antibodies 132. CHO cells are also employed in co-culture techniques with variations in plasmid design, inoculation, and harvesting processes 133.

Given the complexity of producing IgG-like BsAbs, which typically require two plasmids for heterodimerized heavy chains and one for a shared light chain or two separate light-chain plasmids, mammalian cells are predominantly used 43. CHO and KEK293 cell lines are considered optimal for this purpose 134,135.

The design of BsAbs is also influenced by their affinity and valency. Affinity affects the cytokine release, drug distribution, and overall tolerability of BsAbs. For instance, a CD3 arm with high affinity can trigger excessive cytokine release and reduce the ability to target disease sites 136,137. Research has shown that PSMA˟ CD3 BsAbs with lower CD3 affinity are more effective at eliminating tumor cells and reducing the incidence of cytokine release syndrome (CRS) in prostate cancer than BsAbs with higher CD3 affinity 138.

Valency, or the number of antigen binding sites, also plays a role in the efficacy of BsAbs. For example, glofitamab, with a 2:1 valency ratio against CD20 and CD3, exhibits an anti-tumor activity that is 40 times greater than that of BsAbs with a 1:1 valency 139.

## 4. Urological cancers: A brief description

**Figure 3:**
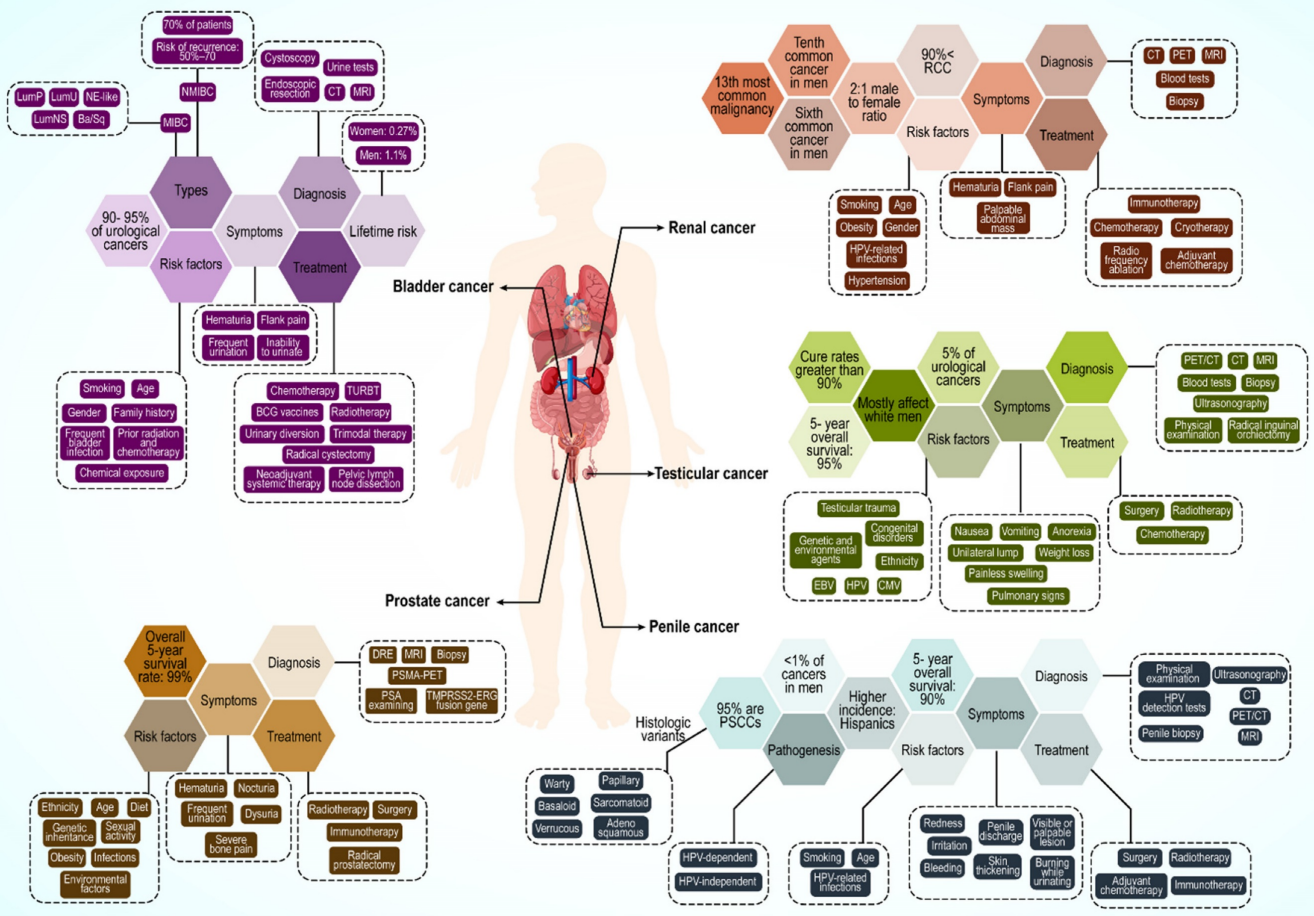
Multiple types of Urological cancers.

Urological cancer is a general term for prostate, kidney, bladder, testicular, and penile cancers (**Figure 3**). Among these, prostate, testicular, and penile cancers are male specific. Prostate cancer is the most common cancer across all racial and ethnic groups. Bladder and kidney cancers are the second and third most common, respectively, whereas penile cancers are very rare. However, this order can change because of racial differences.

### 4.1. Prostate cancer

Prostate cancer remains a significant public health concern for men globally, particularly in Western countries. In the US, prostate cancer is the most common cancer in males (ages 45-60) and a leading cause of cancer-related death across races 140-142. Its global incidence is projected to exceed 1.5 million new cases by 2030 owing to the aging population 143. Black men have a notably higher global incidence.

Prostate cancer originates as an adenocarcinoma of the prostate gland. These cancers can spread beyond the prostate and metastasize to the lymph nodes and bones 144,145. Early stages often lack specific symptoms and potentially mimic benign conditions 146. Metastatic disease commonly presents as intense bone pain in the back, pelvis, hips, or ribs 147. Genetics plays a major role, especially in men with a family history 148. Specific gene mutations (ATM, BRCA1/2, RNase L, MSR1, and ELAC2) and single-nucleotide polymorphisms (SNPs) are major contributors 149,150. Age, obesity, diet, sexual habits, infections, and environmental exposure also influence risk 151-153.

Like other neoplasms, early detection is crucial, and prostate-specific antigen (PSA) testing is a common screening method, with newer biomarkers such as PHI and TMPRSS2-ERG fusion genes gaining traction 154,155. Additional diagnostic tools include digital rectal examination (DRE), biopsy, magnetic resonance imaging (MRI), TRUS, and PSMA-PET scans 156,157.

Treatment depends on the stage and risk. Active surveillance, radical prostatectomy, and radiotherapy are standard treatments for low-risk and stage I-III cancer. Androgen ablation (surgical or pharmacological) is preferred for stage IV and high-risk stage III 158. Sipuleucel-T (Provenge) immunotherapy is used in advanced and hormone-resistant cases 159. Future directions include combination therapies, gene therapies, and strategies to overcome drug resistance 160,161.

### 4.2. Kidney cancer

Kidney cancer is the 13th most common malignancy worldwide, the sixth most common cancer in men, and the tenth most common in women 162,163. Renal cancer mostly occurs in European and North American populations, with a lower incidence in Asian individuals.

More than 90% of kidney cancers are renal cell carcinomas (RCC) originating from renal tubular epithelial cells 164. Other subsets of kidney cancers include urothelial carcinomas, sarcomas, and mesenchymal tumors, which occur at lower rates 165. RCC is usually diagnosed at the age of 50-70 years, with an approximate 2:1 male to female ratio. A continuously increasing incidence has been reported, particularly in developed countries. Mortality has dropped by approximately 1% every year since 2008; however, RCC remains the most lethal urological cancer in comparison to bladder and prostate cancers, with a mortality rate of 30-40% 166,167.

Typical symptoms of RCC include hematuria, flank pain, and a palpable abdominal mass, which is seen in only a small percentage of patients 168. Approximately, 30-50% of patients progress to metastasis with local disease, and 40% with localized RCC have distant metastases involving the lungs, bones, brain, adrenal glands, other kidney, and liver 169.

RCC is a heterogeneous cancer classified based on histological differences that affect the prognosis and treatment choice. Clear cell RCC (70-90%), papillary RCC (10-15%), and chromophore (3-5%) are the most common RCC subtypes. Age and sex are the most important risk factors for RCC 170. Ethnicity, smoking history, use of tobacco products, hypertension (HTN), and obesity are other possible risk factors 171-173.

Approximately 3% of RCC cases have a familial background with an autosomal-predominant pattern. Some of the genes involved in RCC incidence are VHL, MET, FH, BHD, and HRPT2, with most mutations in the VHL gene causing hereditary clear cell RCC 174. In addition to laboratory tests, contrast-enhanced ultrasound (CEUS), computed tomography (CT), MRI, positron emission tomography (PET), and tissue biopsy are used for RCC diagnosis 175,176.

Surgical treatment remains crucial for RCC. Further, active surveillance, cryotherapy, radio frequency ablation, and adjuvant therapy are other available forms of treatment. In metastatic RCC, immunotherapy using immunosuppressive factors such as IFN-α and IL-2 is common. Recently, nivolumab, an anti-PD-1 immune checkpoint inhibitor, has emerged as a promising treatment 177-179.

### 4.3. Bladder cancer

Bladder cancer is among the most prevalent cancers worldwide, ranging from nonaggressive and usually noninvasive tumors to aggressive or advanced-stage disease with high mortality. Approximately 90% of individuals with bladder cancer are older than 55 years 180. The lifetime risk of bladder cancer is approximately 1.1% in men and 0.27% in women. The incidence rate is higher in Western countries, largely because of carcinogen exposure. It is the eighth most common cause of cancer-related deaths in men in the US, but its mortality rate has decreased from 2016 to 2020 181. Bladder cancer is a carcinoma of urothelial cells that accounts for 90-95% of all urothelial carcinomas 182. It can present as non-muscle-invasive bladder cancer (NMIBC), considered as stage Ta and T1, and muscle-invasive bladder cancer (MIBC) or a metastatic form of the disease which includes T2-T4 stages. Furthermore, there is a distinct phenotype of noninvasive lesions with a high rate of recurrence, known as carcinoma *in situ* (CIS) 182,183.

Approximately 70% of patients with bladder cancer have NMIBC, with a high risk of recurrence (50-70%); this progresses to MIBC form in 10-20% of cases 184.

The most obvious symptom of bladder cancer is hematuria, which may only be visible or detectable by microscopy. Other reported symptoms are nonspecific and include frequent urination, pain during urination, flank pain, and inability to urinate 185.

Age is the greatest risk factor for bladder cancer owing to increased exposure to carcinogens and a reduced ability to repair DNA. Other risk factors include smoking and tobacco use, sex, family history, frequent bladder infection, prior radiation and chemotherapy, and chemical exposure 186-189. Men are diagnosed with bladder cancer three to four times more often than women 185. Bladder cancer is a cancer with many mutations, with 70-80% of patients having mutations in the promoter of the gene encoding telomerase reverse transcriptase (TERT) 190. Moreover, in NMIBC cases, the most frequent phenotype, other DNA abnormalities have been noted, such as deletions in chromosome 9 and mutations in genes encoding FGFR3 and PI3K, which could be an early evidence for malignancy 191,192. Strategies for bladder cancer diagnosis include cystoscopy and endoscopic resection, cross-sectional urography (i.e., CT or MRI), urine tests, and urine cytology tests 193-195.

The best method for managing bladder cancer is selected based on the cancer type. In perioperative chemotherapy for patients with NMIBC, Bacille Calmette-Guérin (BCG) vaccines and resection of the bladder tumor (TURBT) are treatment options 196. Neoadjuvant systemic therapy, radical cystectomy, pelvic lymph node dissection, urinary diversion, and trimodal therapy are used to treat MIBC. In the case of metastasis, cytotoxic chemotherapy and immunotherapy, particularly with immune checkpoint inhibitor mAbs against PD-1 and its ligand, PD-L1, are used 197,198.

### 4.4. Testicular cancer

Testicular cancer is a rare disease accounting for 5% of urological cancers and the most common malignant tumor in young men aged 15 to 34 years, with an increasing incidence worldwide in the past two decades 198-200. Excellent outcomes have been reported for testicular cancer, with cure rates greater than 90% for all stages and 95% five-year survival rates 201. Testicular cancer mostly affects white men; therefore, an increase in the incidence rate of the disease is expected in European countries until 2035 202,203.

The transformation of primordial germ cells (PGCs) and formation of germ cell neoplasia in situ (GCNIS) are regarded as precursor lesions to malignant testicular germ cell tumors (TGCTs) 204,205. These can progress to 1) seminoma, 2) embryonal carcinoma cells, 3) choriocarcinomas and yolk-sac tumors, and 4) teratomas. Embryonal carcinoma cells, choriocarcinomas, yolk-sac tumors, and teratomas are also known as nonseminomas 201. Approximately 50% of patients with testicular cancer worldwide are diagnosed with seminoma, whereas the others may have various types of non-seminomas or mixed TGCTs 206.

Testicular cancer typically appears as a unilateral lump, and painless swelling with pain is noted in approximately 10% of the patients. Symptoms or signs of metastatic disease such as weight loss, nausea, vomiting, anorexia, pulmonary and lymphatic involvement (shortness of breath and lymphadenopathy), bulky retroperitoneal disease, vascular obstruction or thrombosis, and gastrointestinal hemorrhage are rarely present in patients 207.

Both genetic and environmental factors are involved in testicular cancer development. Congenital disorders, such as cryptorchidism (one or both testicles absent in the scrotum), hypospadias (the urethral opening not at the head of the penis), and low sperm count, are associated with higher testicular cancer occurrence 208,209. Family history, ethnicity, infections such as human papillomavirus (HPV), Epstein-Barr virus (EBV), and cytomegalovirus (CMV), testicular trauma, and carcinoma in situ are other risk factors 210-212. Anomaly of the short arm of chromosome 12 is pathognomonic for all types of adult germ cell tumors and is an example of genetic risk factors for testicular cancer 213.

Disease evaluation is achieved by physical examination, blood tests (AFP, bHCG, and LDH), CT, PET/CT, MRI, scrotal ultrasonography, radical inguinal orchiectomy, and biopsy 214,215. Germinal cell testicular cancer is managed based on tumor stage, similar to other urological cancers. Treatment strategies include surgery (orchiectomy), radiation therapy, chemotherapy, and stem cell transplantation. Occasionally, more than one type of treatment is used 215.

### 4.5. Penile cancer

Penile cancer is a rare malignancy accounting for less than 1% of cancers in men 216. The incidence of this cancer has been increasing over the past decade but varies between different populations depending on the risk factors 217,218. No significant difference is seen between white and black men; however, a higher incidence is noted among Hispanics 219. Penile cancer is usually diagnosed in men over 60 years of age, but can also manifest in younger patients 220,221.

Approximately 95% of penile cancers are penile squamous cell carcinomas (PSCCs), which can be subdivided into several histologic variants such as basaloid, warty, papillary, verrucous, sarcomatoid, adenosquamous, and other rare types. Basaloid, adenosquamous, and sarcomatoid subtypes are aggressive tumors 222. Moreover, mucosal melanoma, sarcoma, and extramammary Paget's disease can also occur in the penis 223.

Penile cancer pathogenesis can be classified as HPV-dependent- or HPV-independent. In the HPV-dependent type, by integrating HPV DNA into the host genome with expression of the E6 and E7 oncogenes, host cells transform into a malignant phenotype, resulting in malignant HPV-related lesions 224. HPV-related lesions in penile cancer appear as low-grade squamous hyperplasia while high-grade lesions appear as invasive carcinomas of the penis 225.

In HPV-independent penile cancers, a premalignant precursor lesion is involved, which is associated with chronic inflammation. In some chronic penile conditions, including balanoposthitis, phimosis and lichen sclerosus, an increase in cyclooxygenase 2 (COX2) expression drives the overproduction of prostaglandins 226, resulting in activation of EGFR, b-catenin, and PI3K, as key players in carcinogenesis. Furthermore, the loss of heterozygosity in p16, a tumor suppressor gene, is frequently reported in penile cancer 224. Finally, the production of reactive oxygen species (ROS) and reactive nitrogen species (RNS) during persistent inflammation induces DNA damage and genomic instability 227.

Penile cancer symptoms include redness and irritation on the penis, skin thickening, and a visible or palpable lesion manifesting as a painless lump or ulcer on the glans, coronal sulcus, or foreskin. Penile cancer is accompanied by bleeding from the penis, penile discharge, or burning sensation during urinating 228,229.

Smoking is one of the main risk factors for penile cancer 230. Age and some infections such as HPV (especially strains 16, 18, 31and 33) and HIV infection, circumcision status, chronic inflammatory conditions, and poor hygiene are other risk factors for penile cancer 230, 231. Early diagnosis and staging are crucial because lymphatic spread is strongly associated with poor prognosis 232. Physical examination of the penis and inguinal lymph nodes, HPV detection tests, ultrasonography, CT and PET/CT, MRI, and penile biopsy are common methods for diagnosing this cancer type 233,234. Treatment of penile cancer varies depending on the clinical stage. Treatments include surgery, radiotherapy, adjuvant chemotherapy and chemotherapy, and immune checkpoint blockade 229,235,236.

## 5. Targeting BsAbs in urological malignancies

**Figure 4:**
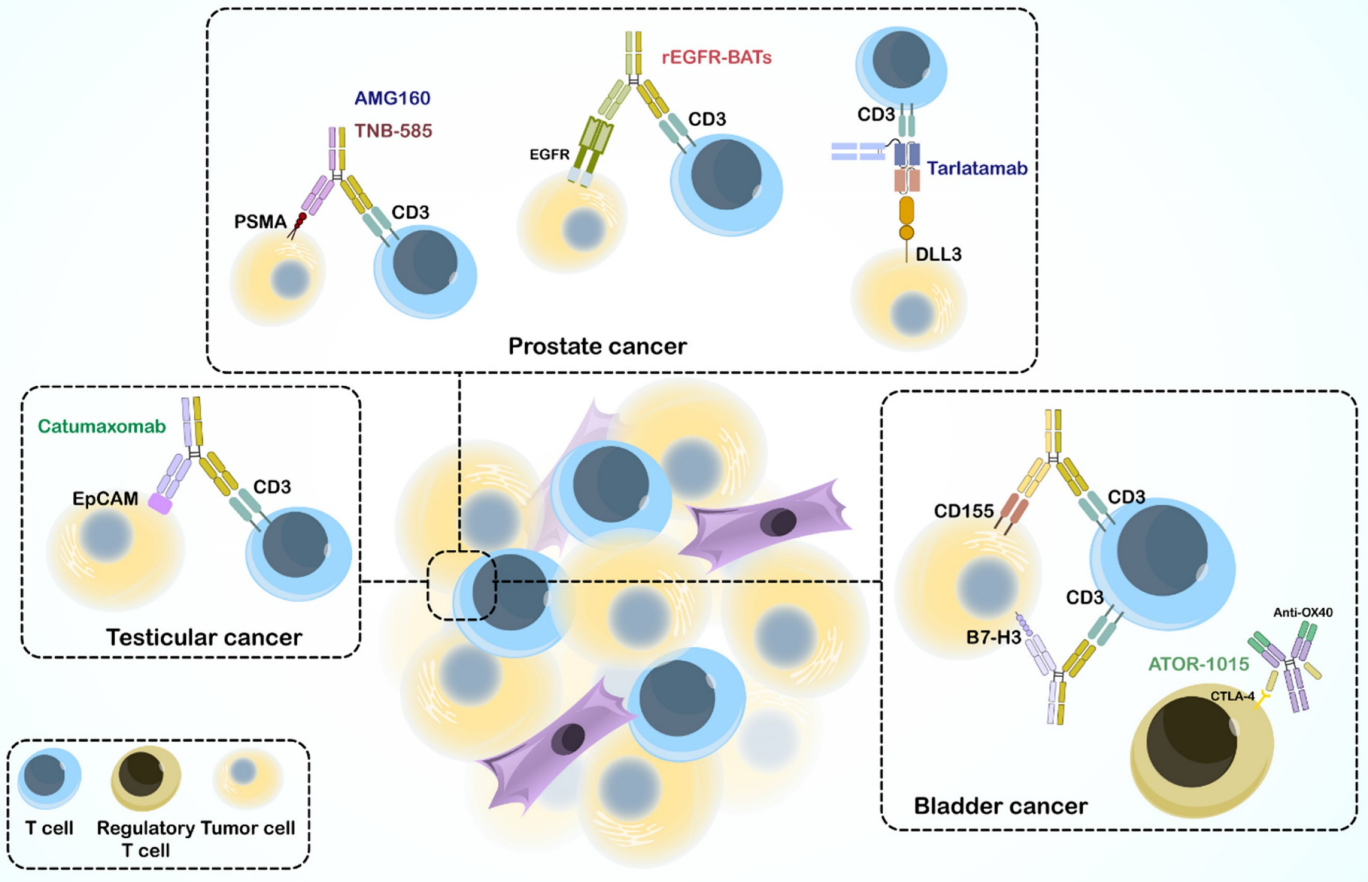
Bispecific antibodies in treating urological cancers.

The immune system, especially cell-mediated immunity, plays a vital role in defense against cancer and cancer prevention. Recent immunotherapeutic drugs, including mAbs, BsAbs, immune checkpoint inhibitors, vaccines, CAR-T cell therapy, and immune cell transfer combat different mechanisms and affect different stages of cancer. Ag-specific immunotherapy stimulates the immune system to recognize tumors that express AgNPs to mediate tumor killing. An interesting focus in immunotherapy is the appearance of engineered BsAbs, which serve as a linker between immune cells and tumors but also many other defensive mechanisms discussed in Section 3, with an increasing ability to overcome drug resistance and decrease adverse effects. Over the past decades, immunotherapy has emerged as a treatment option for various urological malignancies, promising to change the field of urologic oncology. Numerous trials have used BsAbs to treat solid tumors and hematological malignancies. Here, we review the preclinical and clinical studies on BsAbs for specific urological cancers (**Figure 4**).

### 5.1. Preclinical studies using BsAbs in urological cancer models

The challenges posed by the prostate tumor environment have spurred research on novel immunotherapies. BsAbs are emerging as promising tools because of their ability to precisely target immune cells, tumor cells, and cancer-associated pathways 237,238.

Several preclinical studies have highlighted the potential of BsAbs in prostate cancer treatment; 10B3 BsAb, a novel BsAb targeting PSMA and CD3 (in both IgG-like and non-IgG-like forms), has demonstrated significant T cell activation and tumor cell reduction in LNCaP cells (a human prostate cancer cell line). The non-IgG-like form elicited a stronger inflammatory cytokine response, whereas the IgG-like form resulted in complete tumor regression *in vivo* 239. TNB-585 BsAb is another BsAb that combines an anti-PSMA arm with a low-affinity anti-CD3 arm, targeting PSMA+ tumor cells and patient-derived prostate cancer cells. Compared to high-affinity anti-CD3 BsAbs, TNB-585 leads to less cytokine release and regulatory T cell activation, while effectively destroying tumor cells 136-138. AMG160, as a BiTE antibody, has been found to target PSMA and CD3, effectively inducing T cell-mediated cytotoxicity against PSMA-expressing prostate cancer cells in preclinical studies. Encouraged by these results, AMG160 is currently undergoing clinical trials (NCT03792841) 240,241. Another BiTE is AMG 757, also known as tarlatamab, an extended half-life BiTE that targets CD3 and delta-like ligand 3 (DLL3), demonstrated potential in preclinical models for treating neuroendocrine prostate cancer, particularly when DLL3 is overexpressed 242. BC261 BsAb can also target CD3 and STEAP-1, with significant potential for immune cell infiltration and anti-tumor effects in prostate cancer cell lines, suggesting its therapeutic potential 243. Beyond targeting PSMA and CD3, bispecific antibody-armed activated T-cells (BATs) are another promising approach. Huang *et al.* demonstrated enhanced cytotoxicity and anti-tumor activity of T cells armed with a recombinant anti-EGFR and anti-CD3 BsAb 244.

The success of BsAbs extends beyond prostate cancer. Studies have demonstrated their potential in bladder cancer treatment, as ATOR-1015 BsAb, a human CTLA-4 ˟ OX40 IgG-like BsAb, activates T cells, suppresses regulatory T cells, and reduces tumor growth in bladder cancer models 245. BsAb anti-CD3 ˟ B7-H3 BATs have exhibited significant anti-cancer activity against bladder cancer cell lines, including chemoresistant lines 246. Moreover, CD155 Bi-armed activated T cells, as armed T cells, display increased cytotoxic activity and cytokine secretion compared to unarmed activated T cells, suggesting a novel therapeutic strategy 247. Finally, catumaxomab has shown good tolerance and potential efficacy against EpCAM-positive recurrent bladder cancer 248. Furthermore, catumaxomab has demonstrated potential for treating testicular cancer by engaging T cells and promoting NK cell-dependent cytotoxicity against various GCT cell lines 249.

Although these preclinical studies provide a promising picture for BsAbs in urological cancers, further investigations are crucial. Large-scale clinical trials are necessary to determine their efficacy and safety profiles in real-world patients. Additionally, research on optimizing BsAb design and identifying the most effective treatment combinations can unlock their full potential to revolutionize urological cancer treatment.

### 5.2. Clinical trials and treatment landscape in urological cancers

By the end of 2023, a search of the clinicaltrials.gov website revealed over 20 active clinical trials utilizing BsAbs for treating urological cancers, but no BsAbs have been specifically approved by the FDA for this purpose.

BiTEs represent a notable category of BsAbs subjected to both preclinical and clinical evaluation. A phase 1 clinical trial reported at the ESMO meeting in 2020 demonstrated that AMG160 (PSMA ˣ CD3) exerted a disease-suppressive effect in patients with metastatic castration-resistant prostate cancer (mCRPC). PSA levels were reduced by more than 50% from the baseline in 34.3% (12 out of 35) of the cases, and 23.1% of patients experienced disappearance of circulating tumor cells during treatment. However, CRS occurred in 66% of the patients, albeit in a manageable form (NCT03792841) 250.

In another phase 1 clinical trial, CC-1 BsAb combined with tocilizumab, an IL-6 receptor (IL-6R) antagonist—was used prophylactically in 14 patients with prostate carcinoma. Although CRS was observed in 79% of patients treated with IgG-based BsAbs targeting PSMA and CD3, all heavily pretreated patients experienced a rapid decrease in elevated PSA levels (up to 60%). T-cell activation was also observed (NCT04104607) 251,252.

A phase 2 clinical trial assessed the safety and efficacy of HER2Bi-armed activated T cells (HER2 BATs) in combination with pembrolizumab, a PD-1 inhibitor, in mCRPC. This trial was the first comprehensive report to combine a checkpoint inhibitor with targeted T-cell therapy to evaluate its clinical efficacy. The primary endpoint of 6-month progression-free survival (PFS) was met by 5 of the 14 patients, with a median PFS of 5 months and a median survival of 31.6 months. A decrease in PSA levels of 25% or more was observed in six patients, and five patients (38.4%) were progression-free at six months. Post-infusion immunophenotyping showed significantly increased cytotoxic activity in PBMCs (CTL and KN cells) and a marked decrease in the regulatory T-cell population, indicating the efficacy of the pembrolizumab and HER2 BATs combination, and encouraging further investigation (NCT03406858) 253.

Amgen has recently introduced AMG340, an alternative anti-PSMA × CD3 BiTE to AMG160, for use in mCRPC. The phase 1 multi-center study of AMG340 is currently ongoing (NCT04740034). Additional ongoing trials evaluating the efficacy of BsAbs in prostate cancer treatment include a phase 1/2 study of REGN4336 (a PSMA × CD3 BsAb) administered alone or with cemiplimab in mCRPC (NCT05125016) and a phase 1 trial combining JNJ-87189401 (PSMA × CD28) with JNJ-78278343 (KLK2 × CD3) in advanced prostate cancer (NCT06095089). All clinical trials of BsAbs in prostate cancer treatment are summarized in **Table 2**. BsAbs target various kidney cancer markers, particularly renal cell carcinoma (RCC). These include monotherapy with AK104 (cadonilimab), a tetravalent bispecific IgG1 targeting PD-1 and CTLA-4 (NCT06035224), and in combination with axitinib, a tyrosine kinase inhibitor (TKI), in advanced and metastatic RCC (NCT05256472). Other targets include ENPP3 and CD3 via XmAb819 in advanced clear cell RCC (NCT05433142), HER2 and HER3 by MCLA-128 (zenocutuxumab) in patients with RCC or prostate cancer (NCT04100694), and PD-L1 and CD27 via CDX-527, a tetravalent PD-L1 × CD27 IgG1-scFv BsAb in patients with advanced malignancies, including RCC and bladder urothelial carcinoma (NCT04440943).

An ongoing Phase 1 dose-escalation study is investigating the therapeutic potential of catumaxomab, administered as an intravesical instillation, in patients with non-muscle invasive bladder cancer (NMIBC) at high and intermediate risk for progression. Catumaxomab mediates antibody-dependent cellular cytotoxicity against human epithelial tumor cells, including bladder cancer (NCT04819399). Several clinical trials related to various stages of urological cancer have been completed; however, their results are yet to be published. These include a phase 1 and 2 study on the safety of GEN1044 (DuoBody anti-CD3x5T4 BsAb) in patients with malignant solid tumors such as prostate and bladder cancer (NCT04424641), a phase 1 trial of GEM3PSCA in patients with renal and prostate cancer (NCT03927573), and two separate phase 1 studies evaluating the safety of XmAb20717 (BsAb targeting PD-1 and CTLA-4) (NCT03517488) and XmAb22841 (anti-CTLA-4 × LAG-3) monotherapy and in combination with pembrolizumab (NCT03849469) in urothelial carcinoma, renal cell carcinoma, castration-resistant prostate carcinoma, and squamous cell carcinoma of the penis. All related clinical trials are listed in **Table 2**.

## 6. Efficacy and safety profiles of BsAbs in urological cancers

**Figure 5:**
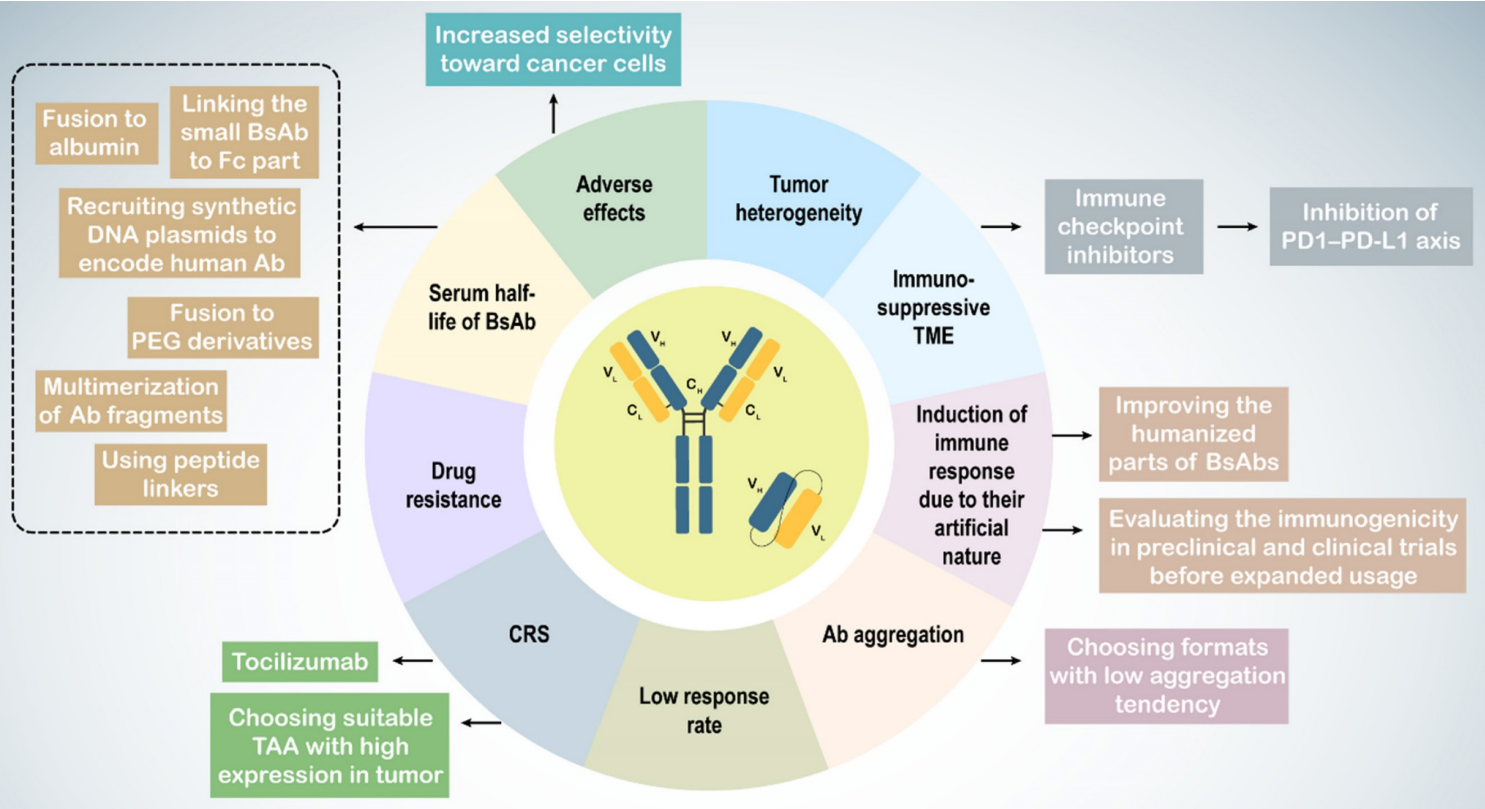
Diagram of efficacy and safety profiles of BsAbs in urological cancers*.*

Clinical trials assessing BsAbs for urological cancer are currently in phases 1 and 2, marking their inaugural use in patient treatment. Typically, first-in-human (FIH) studies aim to determine the safety, tolerability, and initial efficacy of BsAbs in patients with any form of urological cancer (**Figure 5**). These studies generally consisted of two segments: a dose-escalation phase to identify any dose-limiting toxicities, culminating in the establishment of the maximum dose if no such toxicities were observed, and a dose-expansion phase in which additional participants were administered the established maximum tolerated dose (MTD) 251. BsAbs are administered via continuous infusion, and patient safety is closely monitored throughout the process. During the dose-escalation phase, the Data Safety Monitoring Board (DSMB) reviews the safety reports for each patient whose dose has been increased to provide further recommendations or approvals.

The trials were structured around primary and secondary endpoints. The primary endpoints were the incidence and severity of adverse events. Secondary endpoints encompass a range of factors including safety, defined by the incidence and severity of adverse events (CTCAE V.5.0), immunogenicity as measured by the frequency of human-anti-human antibodies (HAHA) in patients, cytokine induction via serum cytokine level measurements, pharmacokinetics reflected in serum BsAb concentrations, anti-tumor activity, changes in tumor markers and responses, survival rates indicated by overall survival (OS) and progression-free survival (PFS), and quality of life as assessed by the European Organization for Research and Treatment of Cancer core quality of life questionnaire scores.

The objective tumor response, or objective response rate (ORR), was determined by the proportion of participants achieving a confirmed complete response (CR) or partial response (PR) according to RECIST 1.1 Criteria 254. In addition to monitoring adverse events, particular attention was paid to adverse events of special interest (AESIs), such as allergic reactions, development of HAHA, and cytokine release syndrome (CRS), with grade 3-5 events classified as severe. The efficacy and safety of BsAbs, which are influenced by structural factors such as affinity, valency, immunogenicity, and the presence or absence of an Fc region, were also evaluated in relation to these secondary endpoints.

In phase 2 trials, the primary goal was to estimate the clinical efficacy of BsAb infusions at specific doses and intervals by measuring the proportion of patients who remained clinically progression-free at predetermined time points post-registration. Secondary goals involve analyzing immune cell phenotypes and functions, cytokine profiles before and after immunotherapy, and their association with clinical outcomes including response rates, PFS, and OS 251,253.

## 7. Challenging and promising solutions in BsAb therapy

Immunotherapy with BsAbs has shown potential for combating tumors in both preclinical and clinical research. However, challenges such as tumor heterogeneity, the tumor microenvironment (TME), Ab immunogenicity, adverse effects including CRS, short serum half-life, low response rates, and drug resistance complicate their use (**Table 3**). Tumor heterogeneity refers to the diverse phenotypic and genotypic characteristics of cancer cells, leading to subpopulations with varying behaviors and responses to immunotherapy.

In urological cancers, particularly prostate cancer, the immunosuppressive nature of the TME weakens immune responses, characterized by an increase in regulatory T cells, myeloid-derived suppressor cells (MDSCs), and tumor-associated macrophages (TAMs) with an M2 anti-inflammatory phenotype, along with the production of anti-inflammatory cytokines and heightened immune checkpoint expression 256,257,270,271. M2 TAMs are associated with higher Gleason grades, increased PSA levels, and poor prognoses in patients with prostate cancer 272. To counteract the immunosuppressive TME and drug resistance, BsAbs may be used alongside other therapies, such as immune checkpoint inhibitors, to enhance immune cell responses 273. Blockade of the PD1-PD-L1 axis improves the activity of blinatumomab and the CD33 × CD3 BiTE AMG330 in acute lymphoblastic leukemia 274,275.

Because of their artificial molecular structure, BsAbs can trigger immune responses, leading to the production of anti-drug antibodies (ADAs) that form BsAb/ADA immune complexes, resulting in drug-related toxicities and reduced efficacy. Factors influencing Ab immunogenicity include BsAb impurities, Ab origin, dosage, and target molecules. Less than 1% of patients treated with blinatumomab develop ADAs 259,260. Strategies to mitigate this issue include enhancing the humanization of BsAbs and assessing their immunogenicity in preclinical and clinical trials to ensure safety and efficacy. Such results have not been reported for BsAbs in urological cancers.

BsAb treatment can cause adverse effects owing to nonspecific T cell activation. CRS, with IL-6 as a critical factor, can be severe or even fatal 276,277. Thus, BsAb therapy can lead to on-target on-tumor activation, which is essential for treatment success, as well as off-target activation, which occurs without target cells, such as when the BsAb antigen-binding site (e.g., anti-CD3 arm) binds with high affinity to its antigen (e.g., CD3), or because of other factors, such as antibody aggregation and Fc receptor (FcR) binding 278.

Slaga *et al.* developed a CD3 × HER2 T-cell-dependent BsAb with low-affinity HER2 arms, demonstrating selective binding to cells with high HER2 expression, reducing adverse effects and improving therapeutic outcomes 261. Alternative administration methods such as subcutaneous injection, may help mitigate these limitations.

Intravenous administration of catumaxomab led to local cytokine release and T cell-mediated hepatotoxicity owing to its active Fc region binding to FcγR-expressing immune cells 279. Engineered Fc domains may reduce FcγR binding in IgG-like CD3-targeting BsAbs. Other approaches include inducing mutations to suppress FcγR binding and using non-IgG-like BsAbs without an Fc region 263,280. Selecting BsAbs with low aggregation tendencies can also address antibody aggregation issues 239.

On-target off-tumor activation, caused by targeting antigens also expressed on non-tumor cells, limits safe dosages and can lead to CRS. Choosing tumor-associated antigens (TAAs) with high tumor expression and low or no expression on normal cells can help overcome this challenge.

Many BsAbs in urological cancer trials target PSMA, which is also expressed at low levels in healthy tissues, such as prostate cells, kidney proximal tubules, and gastrointestinal cells, posing a potential toxicity risk 281,282.

To prevent on-target off-tumor T-cell activation, researchers have utilized the TME characteristic of increased proteolysis. Preclinical studies have designed BsAbs that recruit T cells but are activated via proteolytic cleavage within the tumor, releasing the anti-CD3 binding moiety at the tumor site and avoiding off-target activation and toxicity 264,265. Tocilizumab, a humanized monoclonal antibody against the IL-6R, can be used prophylactically to control CRS 262.

The short serum half-life of non-IgG-like BsAbs presents another clinical challenge 283. Multiple dosing can overcome rapid clearance from circulation. Extended half-life versions of non-IgG-like BsAbs include linking small BsAbs to the Fc part, fusion to albumin, polyethylene glycol (PEG) derivatives, carbohydrates, and dextran, and multimerization of antibody fragments, peptide linkers, and synthetic DNA plasmids encoding human antibodies 74,266,267,284.

*In vivo* production of BsAbs may prevent the rapid renal clearance of non-IgG-like platforms and maintain effective antibody concentrations. Direct gene delivery or inoculation of *ex vivo* genetically modified cells are the two main strategies for *in vivo* production 268,269,285,286. The *in vivo* production of BsAbs has been described in a review by Blanco *et al.* 85.

## 8. Future directions and novel strategies

Research on BsAbs has revealed numerous benefits, including increased specificity and effectiveness. With advancements in their design and manufacture, BsAbs have emerged as pivotal advances in cancer treatment, augmenting traditional therapies. BsAbs are optimally suited for integration into antibody-drug conjugates (ADCs) and chimeric antigen receptor (CAR) T-cell therapies, heralding a potential shift in personalized and combination therapeutic strategies. The advent of multi-specific antibodies, such as tri-specific and tetra-specific variants, can revolutionize cancer immunotherapy. The core principle of ADCs involves coupling the selectivity of BsAbs with the potency of cytotoxic agents, thereby enhancing targeted drug delivery and mitigating side effects. An example is the bispecific HER2 ˟ CD63his-ADC, which recognizes HER2 and CD63, facilitating improved internalization and anti-tumor efficacy of HER2-targeted ADCs. This antibody binds to HER2-expressing cancer cells through its anti-HER2 arm, whereas its second arm attaches to CD63, directing endocytosis-mediated transport of the drug payload directly to tumor cells, resulting in their destruction 125.

Multi-specific antibodies are considered to offer superior therapeutic promise compared to that of BsAbs and mAbs in clinical settings. SAR-442257, a tri-specific antibody, is currently undergoing phase I clinical trials to evaluate its effectiveness in treating relapsed/refractory multiple myeloma (R/R MM) and nonclassical Hodgkin lymphoma (R/R NHL) (NCT04401020). It simultaneously targets CD3, CD28, and CD38, leading to sustained T-cell activation and specific targeting of myeloma cells 287. Additionally, HPN424, another trispecific antibody comprising domains for T-cell engagement, tumor cell targeting, and half-life extension, has demonstrated tolerability and manageable adverse events in phase 1 and 2 clinical trials. A decrease in PSA levels in patients with metastatic castration-resistant prostate cancer (mCRPC) indicates the antitumor activity of HPN424(NCT03577028) 288,289. The first tetraspecific antibody to enter clinical trials was GNC-038, which binds to CD3, CD19, PD-L1, and 4-1BB. Its dual T-cell activation arms and tumor-targeting sites have been assessed for efficacy in lymphomas (NCT05192486) 290.

Addressing cancer, a multifaceted disease, using monospecific monoclonal antibodies (mAbs) often leads to drug resistance and tumor evasion. BsAb-based therapies have shown potential in overcoming these challenges and have shown promising clinical outcomes. However, BsAbs are still in the investigative phase and require further trials to validate the existing data 291.

Future developments in BsAbs will likely involve the identification of more precise targets, novel formats, and innovative combinations of established treatments and other immunotherapeutic approaches.

## 9. Conclusions

Recent breakthroughs in genetic engineering and antibody design have led to the development of BsAbs using various platforms. These platforms allow the creation of BsAbs with different structures and functionalities, primarily focusing on cancer treatment. Success in this field depends on precise design and target selection. BsAbs offer a unique advantage by maintaining the effectiveness of combination therapy while reducing the associated toxicity. Currently, over 200 BsAbs are being evaluated in clinical and preclinical trials, with nine already approved by the FDA for various conditions beyond cancer, including hemophilia A, macular degeneration, and diabetic macular edema.

However, extensive research on BsAbs has revealed several challenges that limit their applications. Overcoming these hurdles begins with an optimal design and rigorous target identification. BsAbs represent a major leap forward in drug development, enabling the development of novel drugs that can simultaneously target multiple pathways. Their high specificity makes BsAbs ideal candidates for antibody-drug conjugates (ADCs) to improve cancer cell elimination. Additionally, BsAbs can be used to activate T cells and potentially boost anti-tumor responses. Exploration of multi-specific antibodies holds significant promise for enhancing the ability to fight cancer. In the context of urological cancers, BsAbs are still in the early stages of clinical trials to determine their optimal dosages and initial clinical effectiveness. Nevertheless, preclinical studies using BsAbs in laboratory models of urological cancers, particularly prostate cancer, have shown positive results, providing a basis for further investigation and development.

## Figures and Tables

**Table 1:** Different subtypes of bispecific antibodies

Format	Date	Company	Design strategy	Ref.
Triomab quadroma	1995	Fresenius and Trion Pharma	Somatic fusion of mouse and rat hybridomas	(27,28)
Knobs-into-holes	1996	Genentech	Forming a knob by substituting a smaller amino acid with a larger one in the CH3 domain of an Ab chain Forming a hole by replacing a larger amino acid in the other chain with a smaller amino acid	(29)
CrossMab	2011	Roche	CrossMabVH-VL format: swapping heavy and light chain domains within one Fab of the Ab CrossMabCH1-CL format: swapping CL of the light chain with the CH1 domain of the heavy chain	(30)
Orthogonal	2014		Indicating mutation in the variable region of Fab in the antibodies	(31)
Duobody		Genmab	Exchanging Fab arm between two antibodies	(32)
XmAb	2019	Xencor, Inc.	Introducing mutations at the CH3-CH3 interface	(33)
DVD-Ig	2007	Abbott	Connecting two variable domains in each Fab arm with short flexible linkers and creating a tetravalent bispecific antibody with four antigen binding sites	(34,35)
FIT-Ig	2017	Epimab Biotherapeutics, Inc.	Similar to DVD-Ig but the FIT-Ig structure contains two pairs of Fab domains	(36)
	
BiTE	1995	Amgen	Two ScFv regions of two monoclonal antibodies bound together by a flexible peptide linker	(37)
DART		Macrogenics	Connecting VL and VH (ScFv) domains of one antibody with the VL and VH (ScFv) of another antibody, forming heterodimer polypeptide chains	(38)
TandAb		Affimed	Pairing two chains in opposite direction to create a tetravalent molecule and each chain has two antigen binding sites	(26)
Bi-Nanobody	2018	Ablynx	The VH domains of two or more antibodies connect together and a bispecific antibody is achieved	(39)

**Table 2:** Clinical trials using bispecific antibodies in urological cancer therapy

Antibody	Targets	Platform/ Format	Sponsor	Type of cancer	Status	Phase	location	Start date	Finishing date	NTC number
CC-1	CD3 PMSA		University Hospital Tüebingen	Castration resistant prostate carcinoma	Recruiting	1	Germany	2019- 11-15	2024- 12-31	NCT04104607
JNJ-87189401 + JNJ-78278343	CD28 PMSA + CD3 KLK2		Janssen Research & development, LLC	Advanced prostate cancer	Recruiting	1	United states	2023- 11-01	2027- 06-21	NCT06095089
BAY2010112 (Pasotuxizumab)	CD3 PMSA	BiTE	Bayer	Castration-resistant prostate cancer	Completed	1	Austria + Germany	2012- 11-02	2018- 09-26	NCT01723475
CC-1	CD3 PMSA		University Hospital Tuebingen	Recurrence of prostate cancer	Recruiting	1	Germany	2022- 11-11	2025-12	NCT05646550
AMG 340	CD3 PMSA	BiTE	Amgen	Metastatic castration-resistant prostate carcinoma	Active, not recruiting	1	United states	2021- 04-29	2024- 09-30	NCT04740034
ES414	CD3 PMSA		Aptevo Therapeutics	Metastatic castration-resistant prostate cancer	Completed	1	United states + Australia	2015-01	2019- 02-18	NCT02262910
GEM3PSCA	CD3 PSCA	ATAC	AvenCell Europe GmbH	Solid tumors including prostate cancer + Renal cancer	Terminated	1	Germany	2019- 04-15	2023- 06-28	NCT03927573
HER2Bi-armed activated T cells	CD3 HER2		Barbara Ann Karmanos Cancer Institute	Metastatic castration resistant prostate cancer	Completed	2	United states	2018- 06-07	2022- 11-07	NCT03406858
GEN1044	CD3 5T4	Duobody	Genmab	Advanced or metastatic solid tumor including prostate cancer + Bladder cancer	Terminated	1/2	United sates + Denmark + Israel	2020- 07-15	2021- 10-29	NCT04424641
XmAb20717	PD-1 CTLA-4	XmAb	Xencor, Inc	Metastatic castration-resistant prostate cancer + Renal cell carcinoma + Urothelial carcinoma + Squamous cell carcinoma of penis	Completed	1	United sates	2018- 07-10	2022- 09-06	NCT03517488
XmAb20717	PD-1 CTLA-4	XmAb	Xencor, Inc	Metastatic castration-resistant prostate cancer	Recruiting	2	United sates	2022- 07-21	2025- 05-30	NCT05032040
REGN4336	CD3 PMSA		Regeneron pharmaceutical	Metastatic castration-resistant prostate cancer	Recruiting	1/2	United sates	2021- 11-30	2026- 08-04	NCT05125016
MCLA-128 (Zenocutuzumab)	HER2 HER3	IgG1-based	Merus N.V.	Metastatic castration-resistant prostate cancer	Recruiting	2	United sates	2022- 11-17	2026-03	NCT05588609
PT217	HuCD47 HuDLL3		Phanes Therapeutics	Advanced refractory cancers including neuro-endocrine prostate cancer	Recruiting	1	United sates	2023-06	2025-06	NCT05652686
XmAb20717 (Vudalimab) Combination therapy	PD-1 CTLA-4		Emory University	Metastatic castration-sensitive prostate cancer	Recruiting	1	United sates	2023- 08-03	2027- 12-16	NCT05733351
XmAb808	CD28 B7-H3	XmAb 2+1	Xencor, Inc	Advanced solid tumors including castration-resistant prostate cancer + Urothelial carcinoma	Recruiting	1	United sates	2022- 12-14	2027-12	NCT05585034
MGC018 ADC (vobramitamab duocarmazine)	CD3 B7-H3		MacroGenics	Advanced solid tumors including castration-resistant prostate cancer + Renal cell carcinoma	Recruiting	1	United sates	2022- 04-19	2025-03	NCT05293496
XmAb22841	LAG-3 CTLA-4	XmAb	Xencor, Inc.	Advanced solid tumors	Completed	1	United states	2019- 05-29	2023- 02-16	NCT03849469
anti-CD3-MUC1 armed CIK	CD3 MUC-1		Fuda Cancer Hospital, Guangzhou	Advanced kidney cancer	Withdrawn	2	China	2018- 04-10	2018- 04-10	NCT03540199
XmAb819	CD3 ENPP3	XmAb	Xencor, Inc.	Clear cell renal cell carcinoma	Recruiting	1	United states	2022- 06-13	2027-03	NCT05433142
SI-B003	PD-1 CTLA-4		Sichuan Baili Pharmaceutical Co., Ltd	Solid tumors including kidney cancer	Recruiting	1	China	2020- 11-10	2023-12	NCT04606472
JNJ-78306358	CD3 HLA-G		Janssen Research & Development, LLC	Advanced solid tumors including renal cell carcinoma	Completed	1	Israel + Spain	2021- 10-24	2023- 02-09	NCT04991740
CDX-527	CD27 PDL-1	IgG1-based	Celldex Therapeutics	Solid tumors including renal cell carcinoma + Bladder urothelial carcinoma	Completed	1	United states	2020- 08-04	2023- 04-06	NCT04440943
AK104 (Cadonilimab)	PD-1 CTLA-4	IgG1-based	RenJi Hospital	Advanced or metastatic clear cell carcinoma	Recruiting	2	China	2023- 08-23	2026- 07-31	NCT06035224
CDX-585	PD-1 ILT4	IgG1-based	Celldex Therapeutics	Advanced malignancies including renal cell carcinoma + Bladder urothelial carcinoma	Recruiting	1	United states	2023- 05-11	2026-02	NCT05788484
MCLA-128 (Zenocutuzumab)	HER2 HER3	IgG1-based	Merus N.V	Advanced NRG1-fusion positive solid tumor including renal cell carcinoma + Prostate cancer	Available		Not provided			NCT04100694
XmAb23104	PD-1 ICOS	XmAb	Xencor, Inc.	Advanced solid tumors including renal cell carcinoma + Urothelial carcinoma	Recruiting	1	United states	2019- 05-01	2025-09	NCT03752398
XmAb22841	LAG-3 CTLA-4	XmAb	Xencor, Inc	Advanced solid tumors including renal cell carcinoma + Prostate carcinoma + Urothelial carcinoma + Squamous cell carcinoma of penis	Completed	1	United states	2019- 05-29	2023- 02-16	NCT03849469
Catumaxomab	CD3 EpCAM	Triomab quadroma	Lindis Biotech GmbH	Non-muscle invasive bladder cancer (NMIBC)	Unknown	1	Germany	2020- 07-07	2023- 05-30	NCT04819399

**Table 3:** Challenges and promising solutions in bispecific antibody-based immunotherapies

Challenge	Definition		Solution	Ref.
Tumor heterogenicity	Distinct phenotypic and genotypic profile of cancer cells		Personalized and combination therapy	(255)
Tumor microenvironment	Immunosuppressive environment such as increased population of regulatory T cells, MDSCs, TAMs, anti-inflammatory cytokine production, immune checkpoint expression		Combination therapy, esp. with immune checkpoint inhibitors	(256-258)
Antibody immunogenicity	Molecular structures of BsAbs are artificial and do not exist in nature		Improving humanized BsAbs and evaluating their immunogenicity in preclinical and clinical studies prior to large-scale usage	(259,260)
Adverse effects (Cytokine release syndrome)	Off-target activation of T cell	The Ag-binding domain of BsAb binds to its target with high affinity	Designing a BsAb with a lower affinity binding site for Ag and alternative administration routes other than intravenous. Prophylactic treatment with tocilizumab	(261,262)
		BsAb aggregation in non-IgG-like formats	Choosing IgG-like BsAbs with low aggregation tendency Prophylactic treatment with tocilizumab	(261-263)
		Binding of the Fc part of IgG-like BsAbs to the Fc receptor of immune cells or Kupffer cells	Using engineered Fc domains and indicating mutations to suppress FcγR binding Consuming non-IgG like BsAbs instead Prophylactic treatment with tocilizumab	(239,262)
	On-target off-tumor activation of T cells, because of targeting Ags which also have high expression on non-tumor cells		Selecting a TAA with high expression in tumor and no/low expression in normal cells Intratumor proteolytic cleavage of anti-CD3 binding moiety Prophylactic treatment with tocilizumab.	(262,264,265)
Serum half-life	Rapid clearance of non-IgG-like BsAbs from circulation		Fusion of non-IgG-like BsAbs to the Fc part, albumin, or PEG derivatives Multimerization of BsAb fragments Use of peptide linkers Recruitment of synthetic DNA plasmids to encode human BsAb *In vivo* production of BsAbs	(262,266-269)
Drug resistance	Tumor escape from targeted therapy		Combination therapy	(258)

## Abbreviations

Ab: antibody
ADC: antibody drug conjugates
ADCC: antibody-dependent cell-mediated cytotoxicity
ADCP: antibody-dependent cellular phagocytosis
AESI: adverse events of special interest
Ag: antigen
BBB: blood-brain barrier
BCG: Bacille Calmette-Guérin
BiKE: Bispecific killer cell engager
BiTE: bispecific T cell engager
BsAb: bispecific antibody
CAR T-cell: chimeric antigen receptor T cell
CDC: complement-dependent cytotoxicity
CDR: complementarity-determining region
CEUS: contrast-enhanced ultrasound
cFAE: controlled Fab arm exchange
CiTE: checkpoint inhibitory T-cell engager
COX2: cyclooxygenase 2
CR: complete response
CRS: cytokine release syndrome
CTL: cytotoxic T lymphocytes
CTLA-4: cytotoxic T lymphocyte antigen 4
DART: dual-affinity retargeting molecule
DCT: distal connecting tubules
DRE: digital rectal examination
DSMB: data safety monitoring board
DVD-Ig: daul-variable-domain immunoglobulin
EGFR: epidermal-growth factor receptor
EMA: european medicines agency
EpCAM: Epithelial cell adhesion molecule
Fab: fragments of antigen-binding
Fc: crystallizable fragment
FcRs: Fc receptors
FIH: first in human
FIT-Ig: Fabs-in-tandem immunoglobulin
FDA: Food and Drug Administration
GEA: gastroesophageal adenocarcinoma
GCNIS: germ cell neoplasia in situ
HAHA: human-anti-human-Abs
HLE-BiTE: half-life extended BiTE
ICIs: immune checkpoint inhibitors
LAG3: lymphocyte activation gene 3
mAb: monoclonal antibody
mCRPC: metastatic castration-resistant prostate cancer
MDSC: myeloid-derived suppressor cell
MHC: major histocompatibility complex
MRI: magnetic resonance imaging
MTD: maximum tolerated dose
NMIBC: non-muscle invasive bladder cancer
NSCLC: non-small cell lung cancer
ORR: objective response rate
OS: overall survival
PD-1: programmed cell death 1
PD-L1: programmed cell death 1 ligand 1
PHI: prostate health index
PSA: prostate-specific Ag
PSMA: prostate-specific membrane antigen
PSMA-PET: prostate-specific membrane antigen positron emission tomography
RA: rheumatoid arthritis
RCC: renal cell carcinoma
PFS: progression free survival
PGC: primordial germ cell
PR: partial response
PSCC: penile squamous cell carcinoma
RECIST: response evaluation criteria in solid tumors
R/M CC: recurrent or metastatic cervical cancer
R/R ALL: refractory precursor B-cell acute lymphoblastic leukemia
R/R MM: relapsed/refractory multiple myeloma
R/R NHL: relapsed/refractory non-classical Hodgkin's lymphoma
RTK: receptor tyrosine kinases
scFv: single-chain variable fragment
TAA: tumor-associated Ag
TAM: tumor associated macrophage
TandAb: Tandem diabody
T-BsAb: T cell engaging bispecific antibodies
TERT: telomerase reverse transcriptase
TIM3: T-cell immunoglobulin mucin 3
TME: tumor microenvironment
TRUS: transrectal ultrasound
TNFRSF: TNF receptor superfamily
VEGF: Vascular-endothelial-growth factor
